# Dynamic Changes in the Global MicroRNAome and Transcriptome Identify Key Nodes Associated With Ovarian Development in Chickens

**DOI:** 10.3389/fgene.2018.00491

**Published:** 2018-10-23

**Authors:** Wenwen Wang, Keliang Wu, Meiting Jia, Shuhong Sun, Li Kang, Qin Zhang, Hui Tang

**Affiliations:** ^1^Shandong Provincial Key Laboratory of Animal Biotechnology and Disease Control and Prevention, Shandong Agricultural University, Taian City, China; ^2^College of Animal Science and Technology, China Agricultural University, Beijing, China

**Keywords:** chicken, ovarian development, miRNA profiling, mRNA profiling, integrative analysis

## Abstract

The analysis of gene expression patterns during ovarian follicle development will advance our understanding of avian reproductive physiology and make it possible to improve laying performance. To gain insight into the molecular regulation of ovarian development, a systematic profiling of miRNAs and mRNAs at four key stages was conducted, using ovarian tissues from hens at 60 days of age (A), 100 days (B), 140 days-not yet laying (C), and 140 days-laying (D). Comparisons of consecutive stages yielded 73 differentially expressed miRNAs (DEMs) (14 for B vs. A, 8 for C vs. B, and 51 for D vs. C) and 2596 differentially expressed genes (DEGs) (51 for B vs. A, 20 for C vs. B, and 2579 for D vs. C). In addition, 174 DEMs (22 for C vs. A, 74 for D vs. A, and 78 for D vs. B) and 3205 DEGs (118 for C vs. A, 2284 for D vs. A, and 2882 for D vs. B) were identified between nonconsecutive stages. Some DEGs are involved in the Wnt and TGF-beta signaling pathways, which are known to affect ovarian development and ovulation. An integrative analysis of the miRNA and mRNA profiles identified 3166 putative miRNA-mRNA regulatory pairs containing 84 DEMs and 1047 DEGs. Functional annotation of the networks provides strong evidence that the miRNA regulatory networks may play vital roles in ovarian development and ovulation. Ten DEMs and 10 genes were validated by real-time quantitative PCR. The candidate miRNA-mRNA pairs gga-miR-200a-3p-*SFRP4*, gga-miR-101-3p-*BMP5*, gga-miR-32-5p-*FZD4*, and gga-miR-458b-5p-*CTNNB1* potentially associated with ovarian development.

## Introduction

Decades of traditional selective breeding by the poultry industry have resulted in marked improvement in commercial egg-laying breeds (Johnson et al., 2015). The recent development of high-throughput genotyping platforms is likely to enable additional gains in egg production traits through molecular breeding, especially in the indigenous chicken breeds found in developing countries.

Ovarian development and folliculogenesis in chicken is a complex and highly coordinated process, resulting in the maturation of oocytes and the differentiation and proliferation of granulosa and theca cells (Qin et al., 2015; Lyu et al., 2016). Since this biological program is expected to exert a major influence on egg production traits, the cell signaling and associated transcriptional mechanisms that regulate ovarian development are of particular interest. A wide variety of local intraovarian molecular regulation factors participate in folliculogenesis, growth, and development of the ovarian follicles, such as *BMP15*, *STAR*, *CCND2*, *CYP11A1*, *SAV1*, and *GDF9*. Members of the glycoprotein hormone family of gonadotropins are also involved, such as FSH and FSHR (Dong et al., 1996; Bentsi-Barnes et al., 2010; Persani et al., 2014; Zhu et al., 2015). Cell signaling systems like the Hippo/MST signaling pathway are part of the developmental process as well (Pisarska et al., 2010). During the laying period, follicles are recruited into the preovulatory hierarchy from a cohort of small (6-8mm) yellow follicles approximately once a day, a process termed follicle selection (Johnson, 2015). The selected follicle develops rapidly through five (F5 to F1) stage prior to ovulation (Johnson and Woods, 2009).

MicroRNAs (miRNAs) function as reversible regulators of transcription and translation (Cannell et al., 2007) and contribute to at least 60% of the human transcriptome. (Di and Croce, 2013). They have been implicated in a wide range of biological and metabolic processes, including organogenesis, development, cell proliferation, differentiation, and apoptosis, as well as in many diseases (Bartel, 2004; Alvarezgarcia and Miska, 2005; Kloosterman and Plasterk, 2006). Because ovarian development and egg production are a complex traits regulated by many genes, it would be not surprising if they are also regulated through specific miRNA–mRNA interactions. Although some transcriptome studies have focused on mRNA and miRNA expression, the molecular mechanisms involved in the regulation of ovarian development at different stages remains unclear (Kang et al., 2013; Zhu et al., 2015; Wang et al., 2017). High-throughput sequencing of mRNAs and miRNAs together offers a strategy to identify additional genes involved in ovarian development and to understand their interactions with miRNAs in detail.

The present study focused on identifying DEGs and miRNAs at four critical time points during ovarian development in Jining Bairi hens, an indigenous chicken breed that is well known for early sexual maturity at around 100 days. Using the Illumina MiSeq/HiSeq^TM^2000 high-throughput sequencing platform, we analyzed miRNA and gene expression in ovarian tissues at ages of 60, 100, 140 days (but not yet laying eggs), and 140 days (laying eggs). A wide variety of ovarian development-related genes were identified. After comparing expression among the four groups, stage-specific and DEGs were matched to corresponding regulatory pathways by GO and KEGG analyses. mRNA and miRNA interaction networks were also predicted based on the integrated analysis of mRNA and miRNA expression profiles.

## Results

### miRNA Expression Profiling and Screening for Differentially Expressed miRNAs

In order to visualize the pattern of miRNA expression during ovarian development, we conducted a time-course analysis using four samples, obtained at 60 days (A), 100 days (B), 140 days (egg laying has not yet begun; C), and 140 days (egg laying has commenced; D). 8,275,891, 7,091,297, 9,022,100, and 10,724,020 raw reads were collected from the four libraries, respectively. After discarding low quality reads, reads containing 3′ and 5′ adaptors, and reads with other defects, over 97% of the sRNA reads were retained in each of the data sets (Supplementary Table S1). Length distributions of the cleaned reads varied slightly (Supplementary Figure S1), with 21–24 nt and 22 nt as the most abundant lengths in all libraries, consistent with previous results (Kang et al., 2013).

Clean reads were mapped to the NCBI chicken reference genome (galGal4; NCBI) using BowTie. The fraction of mapped reads varied from approximately 46 to 55% per library (Supplementary Table S2 and Supplementary Figure S2). After redundant reads (rRNA, tRNA, snRNA, and snoRNA) were excluded, 507 known miRNAs and 35 novel miRNA candidates were identified by alignment to the miRBase and Rfam databases. 399, 383, 407, and 372 known miRNAs, and 24, 21, 26, and 22 novel miRNAs, were detected in the stage A, B, C, and D libraries, respectively (Supplementary Tables S3, S4).

**FIGURE 1:**
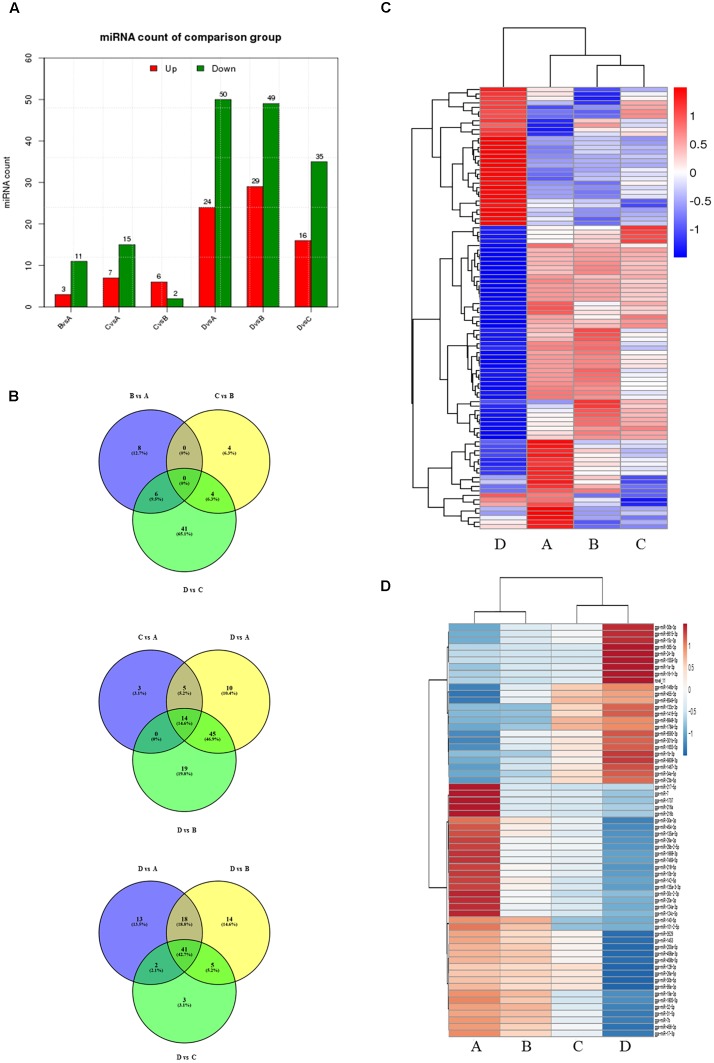
Comparison of miRNA expression at four developmental stages. **(A)** The number of differentially expressed miRNAs (DEMs) in pairwise comparisons between stages. “Up” and “Down” refer to RNAs that are expressed at higher and lower levels in the later of the two stages, respectively. **(B)** Venn diagrams showing commonly and uniquely expressed miRNAs between sequential (top) and non-sequential (middle) stage samples. The diagram at the bottom highlights differences between stage D and the other three stages. **(C)** Hierarchical cluster analysis for miRNA expression at all four stages. **(D)** Hierarchical cluster analysis showing DEMs having sustained decreased/increased expression levels across stages A through D.

To evaluate the development-dependent miRNA activities, we performed a time course differential miRNA expression analysis by comparing temporally adjacent stages after normalizing the mapped reads (Wang et al., 2010) and applying two filtering criteria, *q-*value < 0.01 and |log_2_ (fold change)| > 1 (Supplementary Figure S3). The results are summarized in Figure 1A and detailed in Supplementary Table S5. Comparisons between the four consecutive stages (B vs. A, C vs. B, and D vs. C) revealed 73 DEMs, with 25 miRNA levels decreasing and 48 increasing. Similarly, pairwise comparisons between nonconsecutive stages (C vs. A, D vs. A, and D vs. B) yielded 174 DEMs, of which 60 were decreasing and 114 were increasing. No DEMs were shared by all three consecutive comparisons (Figure 1B, top), while 14 DEMs were shared by the nonconsecutive comparisons (Figure 1B, middle). Stage D samples exhibited a distinct miRNA expression pattern (Figure 1C) and contained the most DEMs when compared with other samples (Figure 1A).

24 miRNAs with sustained increasing expression and 38 miRNAs with sustained decreasing expression in the four consecutive samples were also identified using standardized reads data (Figure 1D).

### Expression Profiling of Ovarian Development-Dependent Genes

**FIGURE 2:**
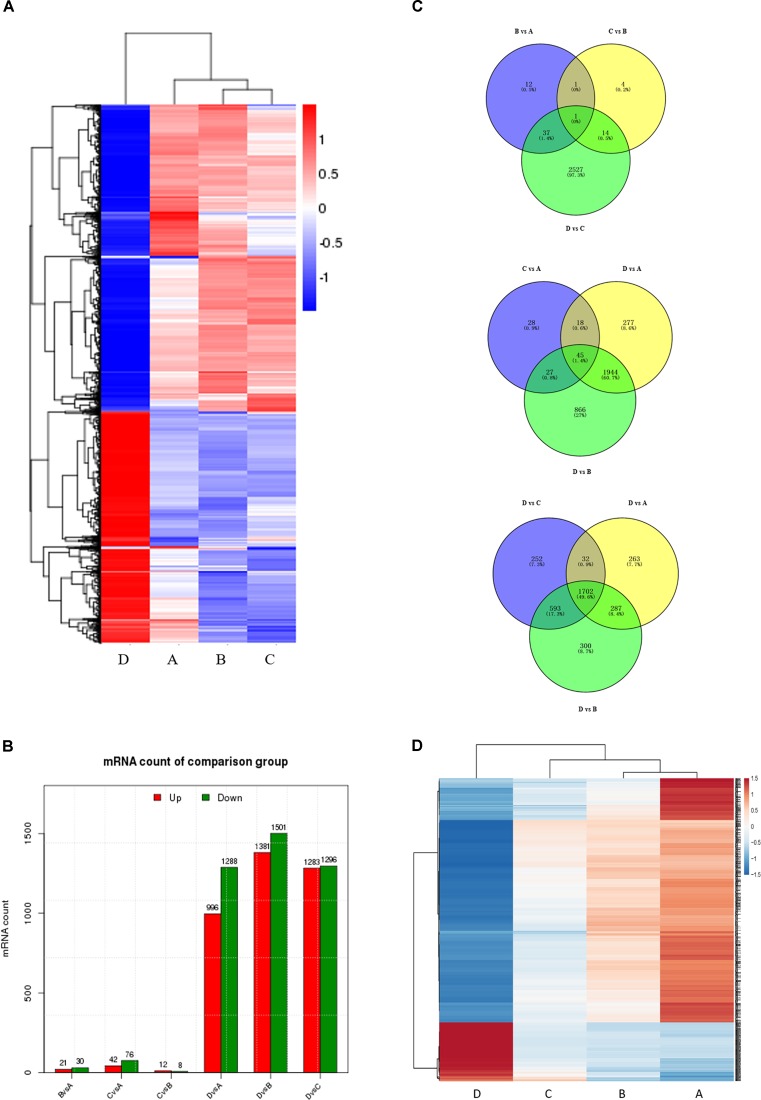
Comparison of mRNA expression at four developmental stages. **(A)** Hierarchical cluster analysis for mRNA expression at all four stages. **(B)** The number of differentially expressed mRNAs (DEGs) between sequential (top) and non-sequential (middle) stages. “Up” and “Down” refer to RNAs that are expressed at higher and lower levels in the later of the two stages, respectively. **(C)** Venn diagrams showing commonly and uniquely expressed DEGs between sequential (top) and non-sequential (middle) stage samples. The diagram at the bottom highlights differences between stage D and the other three stages. **(D)** Hierarchical cluster analysis showing mRNAs having sustained increased/decreased expression levels across stages A through D.

To correlate miRNA expression with gene expression, we analyzed transcriptional changes in the ovaries at four developmental stages in chickens using high-throughput mRNA sequencing (for bulk sequencing statistics, quality control, and assembly results, see Supplementary Table S6 and Supplementary Figure S4). Hierarchical cluster analysis shows that the gene expression profiles differ greatly (Figure 2A), with dramatic mRNA expression changes accompanying the transition to egg laying (stage D). Notably, a very significant proportion of DEGs at all four developmental stages are novel. As was the case for the miRNAs, comparisons between non-sequential developmental stages revealed larger numbers of differently expressed mRNAs (3205) than sequential pairwise comparisons (2596) (Figure 2B and Supplementary Figure S5). Comparisons between the four consecutive time points (B vs. A, C vs. B, and D vs. C) reveal that only 1 differentially expressed mRNAs was common to all three comparisons (Figure 2C, top). In contrast, 45 differentially expressed mRNAs were common to the three nonconsecutive time point comparisons (C vs. A, D vs. A, and D vs. B) (Figure 2C, middle), similar to the bias observed when miRNA expression was compared between time points (cf. Figure 1B). 1702 DEGs were in common in comparisons between sample D vs. A, B, and C (Figure 2C bottom). Of these, some were well-characterized genes involved in follicular development, such as *WNT4* (Hernandez-Gonzalez et al., 2006), *FZD1* (Hsieh et al., 2002), and *FZD4* (Ricken et al., 2002). 133 genes with sustained increased expression, and 553 genes with sustained decreased expression, were identified (Figure 2D and Supplementary Table S7). A complete list of differently expressed mRNAs is provided in Supplementary Table S8.

**FIGURE 3:**
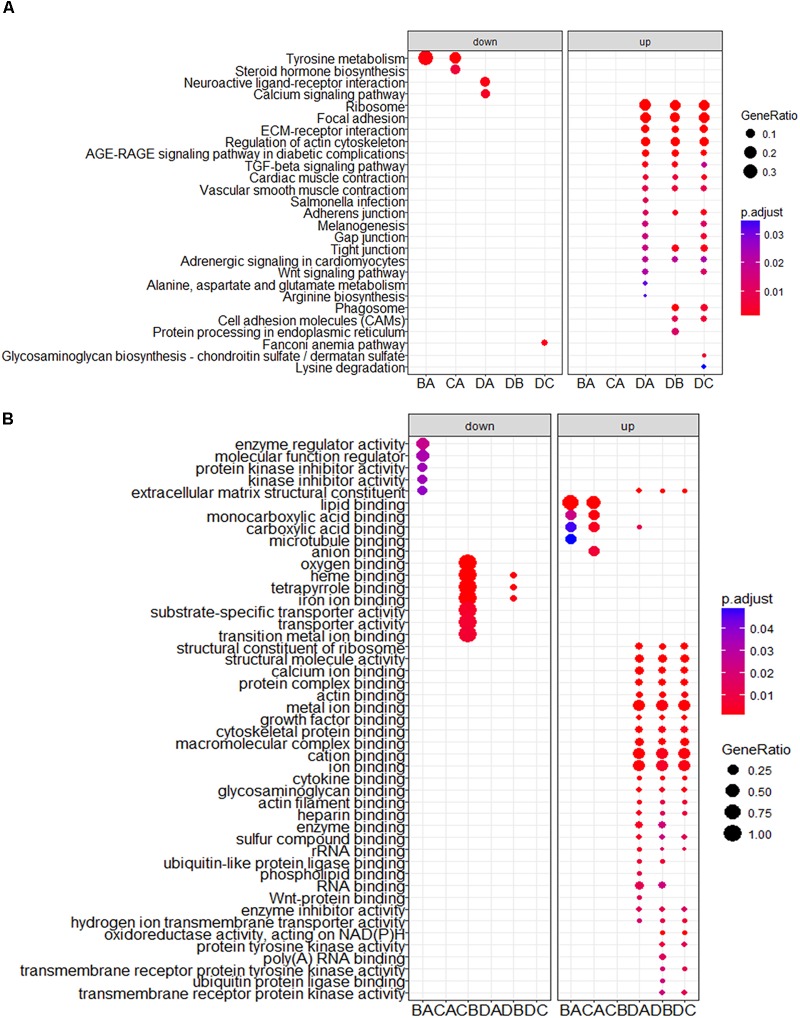
KEGG and GO analysis for DEGs between stages. The top categories obtained by **(A)** KEGG and **(B)** GO term analyses for genes with significantly increased and decreased expression. BA: stage B vs. A; CA: stage C vs. A; DA: stage D vs. A; DB: stage D vs. B; DC: stage D vs. C.

**FIGURE 4:**
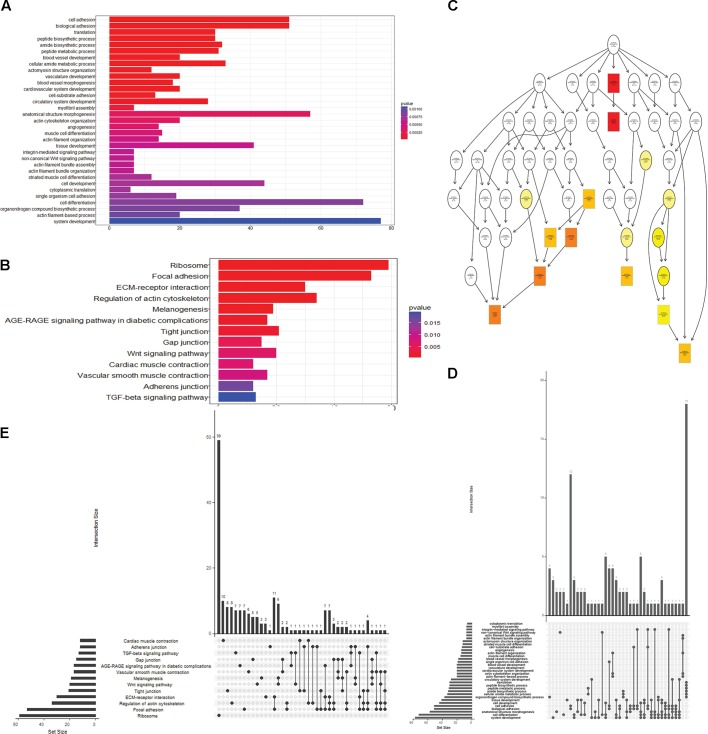
Functional analysis of DEGs between stage D and other stages. **(A)** GO terms and **(B)** pathways that were significantly enriched in the 1702 overlapping DEGs between stage D and the other three stages. The Wnt signaling pathway is highlighted in both panels. **(C)** GO based graph constructed using DEGs associated with the GO term biological process. **(D,E)** Genes belonging to multiple GO term categories **(D)** and KEGG pathways **(E)**.

To obtain a better understanding of DEG functional roles, we performed a gene over-representation test (Boyle et al., 2004) using KEGG and GO for biological theme comparison (Figure 3). Several follicular growth- and ovulation-related pathways and GO terms were identified. For example, expression of components of the Wnt signaling pathway (gga04310) (Boyer et al., 2010), the TGF-beta signaling pathway (gga04350) (Fan et al., 2010), and the Wnt-protein binding biological process (GO: 0017147) increased significantly in stage D chickens. Other pathways such as Ribosome (gga03010), Focal adhesion (gga04510) and ECM-receptor (gga04512) were also enriched in genes with increased expression at stage D, suggesting that these pathways function in follicle development. In order to categorize the genes whose expression changed after the onset of egg laying (stage D), we conducted GO and KEGG analyses for the 1702 DEGs common to stage D and the other stages. Altogether, 91 significant GO terms and 13 pathways were associated with follicular development, including the Wnt signaling pathway (gga04310) and TGF-beta signaling pathway (gga04350) (Figures 4A,B and Supplementary Table S9). To summarize and visualize the findings, we constructed a graph using the GO biological process terms. As shown in Figure 4C, genes are primarily associated with the GO_BP terms translation and system development. We also analyzed complex situations in which a gene belongs to multiple annotation categories (Figures 4D,E). The results show that some genes in the Focal adhesion (gga04510) pathway also participate in the regulation of the actin cytoskeleton (gga04810) and the ECM-receptor interaction process (gga04512). Some genes in the Wnt signaling pathway (gga04310) were also found in Melanogenesis (gga04916) and the TGF-beta signaling pathway (gga04350). Overall, the transition to egg laying has a profound impact on gene expression in the chicken ovary.

### Identification of miRNA–mRNA Regulatory Interactions Associated With Ovarian Development

The integrated analysis of miRNA and mRNA expression profiles should make it possible to identify functional miRNA-mRNA interaction pairs involved in specific biological processes. We applied a multi-step approach to identify and characterize the dynamically co-regulated miRNA-mRNA network related to ovarian development. First, DEMs and their potential targets were investigated based on sequence complementarity and the free energy of the predicted RNA duplex. Since most miRNAs negatively regulate the expression of their target mRNAs by RNA cleavage, we then searched for negative relationships between the levels of miRNAs and their predicted targets, as most studies have done (Wang et al., 2012; Zhang et al., 2016). However, these statistical relationships are not necessarily causal, and can also be the result of the mRNA regulating the miRNA, or a third molecule regulating both the miRNA and the mRNA. To tackle this problem, we applied the R script miRCausality, which implements the IDA method to capture causal regulatory relationships from expression data (Le et al., 2013). After combining pairs having negatively related expression levels with the regulatory effects calculated by miRCausality (*ef* < 0.9), we identified 3166 putative miRNA–mRNA regulatory pairs containing 84 DEMs and 1047 DEGs (Supplementary Table S10).

**FIGURE 5:**
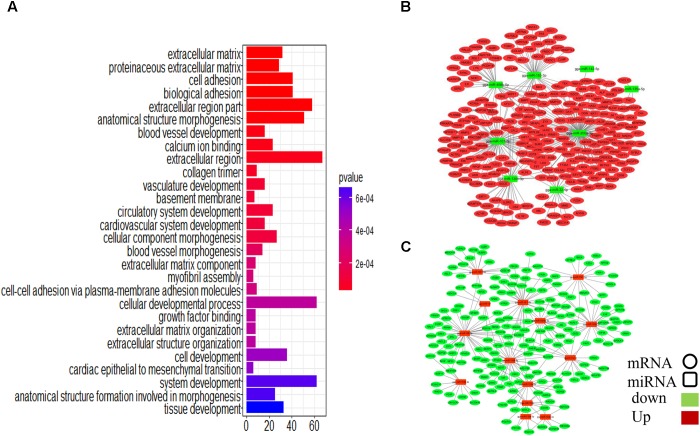
Integrated microRNA/mRNA network analysis. Combining the negatively regulated relationships with regulatory effects (*ef* < 0.9), we identified 3166 putative miRNA-mRNA regulatory pairs, comprising 84 DEMs and 1047 DEGs. **(A)** GO enrichment analysis for DEGs. **(B)** Interaction network constructed using miRNAs with reduced expression. **(C)** Interaction network constructed using miRNAs with increased expression.

To assess the functions of the putative miRNA targets, we conducted a GO enrichment analysis. The results showed that the targets are associated with cell development and tissue development (Figure 5A), similar to the results obtained in the whole transcriptome GO analysis, implying that these miRNAs define a core regulatory network for ovarian development. Key miRNAs were used to nucleate the ovarian development miRNA/mRNA interacting networks shown in Figures 5B,C, and the topologies were analyzed using the NetworkAnalyzer Cytoscape plugin (see Supplementary Table S11).

Some miRNA–mRNA interactions predicted here have already been experimentally confirmed, validating our methods. For example, *FBN2* (Varambally et al., 2008) and *SLC38A2* (Hafner et al., 2010) are targets of miR-101-3p, and *ERBB2IP* is the target of miR-200a-3p (Hafner et al., 2010). However, several novel interactions involved in follicular development-related pathways were also predicted (Table 1). *CTNNB1*, for example, is involved in the Wnt signaling pathway and was identified as a potential target of gga-miR-458b-5p. *BMP5*, which participates in the TGF-beta signaling pathway, was the predicted target of gga-miR-101-3p, gga-miR-135a-2-3p, and gga-miR-153-3p.

**Table 1:** miRNAs and predicted targets involved in follicular development-related pathways.

Pathway	Genes	miRNAs
Wnt signaling pathway	ASPM,FERMT2,CFC1,LATS2,FRZB,PTEN,TCF7L2,SULF1,ILK, SDC1,DKK3,WLS,SFRP4,TLE4,FGFR2,TBL1X, EGR1,MDFIC,SNAI2,MYC,FZD4,FZD3,FGFR3,APCDD1,GP,C3, CTNNB1,LGR4,MDFI,WNT10A,CAPRIN2, BAMBI,NOTCH1,CCNE1	miR-101-3p,miR-130a-3p,miR-135a-2-3p,miR-153-3p,miR-200a-3p,miR-202-5p,miR-29a-3p,miR-30c-5p,miR-30e-3p,miR-32-5p,miR-1747-5p,miR-1a-3p,miR-21-3p,miR-6615-3p,miR-458b-5p,miR-449c-3p,miR-106-5p,miR-125b-5p,miR-3538,miR-99a-5p,miR-138-1-3p,miR-31-3p,miR-29c-3p,miR-31-5p,miR-6660-3p,miR-365-3p,novel_25,miR-449a,miR-6700-3p,miR-1684a-3p,miR-1306-5p,miR-130c-3p,miR-29b-3p,miR-155,miR-204,miR-32-3p,miR-449c-5p,miR-449b-5p,miR-26a-5p,miR-458a-3p
TGF-beta signaling pathway	FST,BMP5,SMAD6,ACVR1,ROCK1,TGFB3,MADH2, THBS1,MYC,ID2,AMH,CHRD,BAMBI	miR-101-3p,miR-130a-3p,miR-153-3p,miR-200a-3p,miR-200a-5p,miR-30e-3p,novel_11,miR-135a-2-3p,miR-106-5p,miR-187-3p,miR-202-5p,miR-30c-5p,miR-204,miR-449c-3p,miR-449b-5p,novel_25,miR-1306-5p,miR-31-5p,miR-29a-3p,miR-29b-3p,miR-29c-3p,miR-130c-3p,miR-458a-3p,miR-32-5p,miR-30e-5p,miR-6660-3p,miR-458b-5p,miR-3525,miR-3529


Four miRNAs, gga-miR-200a-3p, gga-miR-101-3p, gga-miR-32-5p, and gga-miR-458b-5p, have relatively high expression levels across all samples and are highly connected within the miRNA–mRNA network. They are predicted to regulate several genes related to follicular development.

### Validation of miRNA and mRNA Expression Levels Using qRT-PCR

**FIGURE 6:**
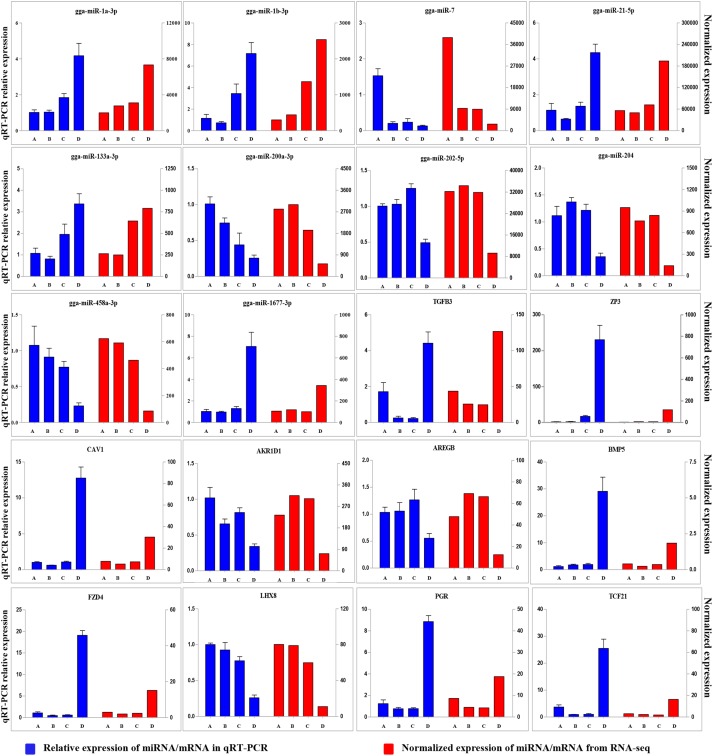
Comparison of expression levels for 10 miRNAs and 10 mRNAs determined using qRT-PCR and RNA-seq. Four samples at each stage were pooled for RNA-seq and four chickens from an independent cohort of animals were used for qRT-PCR. qRT-PCR values are shown as mean ± SD of four measurements, using U6 snRNA and *GAPDH* as internal standards.

**FIGURE 7:**
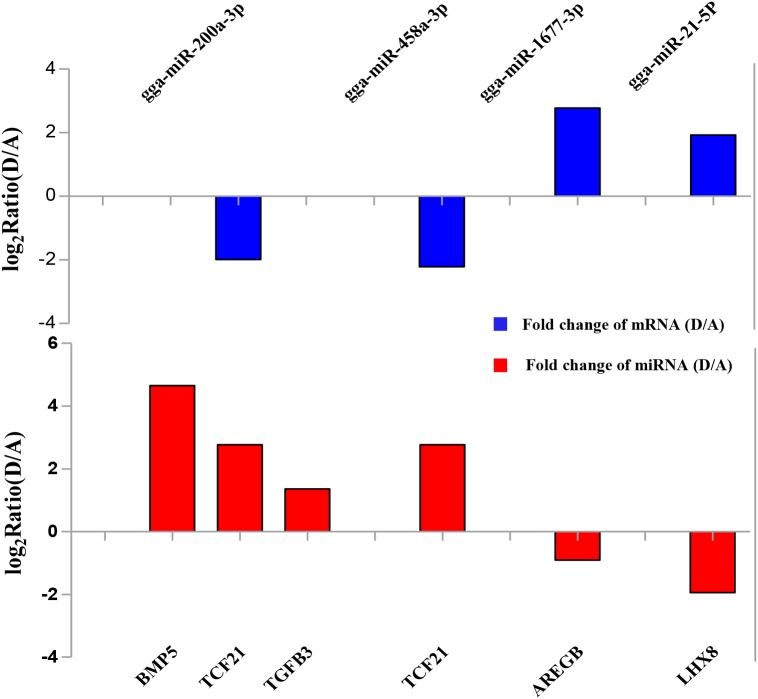
Verification of expression patterns of miRNA-mRNA interaction pairs by qRT-PCR.

**FIGURE 8:**
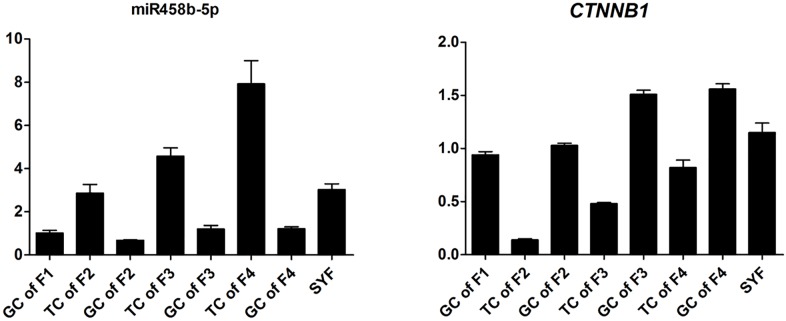
Expression levels of miRNA-458b-5p and *CTNNB1*. GC, granular cells; TC, theca cells; F1, F1 follicles; F2, F2 follicles; F3, F3 follicles; F4, F4 follicles; SYF, small yellow follicles.

To validate the Illumina sequencing data and bioinformatics analyses, we randomly selected 10 miRNAs (gga-miR-1a-3p, gga-miR-1b-3p, gga-miR-7, gga-miR-21-5p, gga-miR-133a-3p, gga-miR-200a-3p, gga-miR-202-5p, gga-miR-204, gga-miR-458a-3p, and gga-miR-1677-3p) and 10 mRNAs (*TGFB3*, *ZP3*, *CAV1*, *AKR1D1*, *AREGB*, *BMP5*, *FZD4*, *LHX8*, *PGR*, and *TCF21*) and measured their levels by qRT-PCR using samples obtained from an independent cohort of animals. The results were consistent with the changes in expression levels determined using RNA-seq data (Figure 6). Consistent results were also obtained for six miRNA–mRNA interaction pairs (Figure 7). We conclude that the miRNA and mRNA sequencing data provide an accurate measure of differences in expression across our four samples. The miRNA-458b-5p/*CTNNB1* pair was validated by qRT-PCR in different cell types and developmental stages using tissues from four hens (granular cells from F1 follicles, theca cells from F2 follicles, granular cells from F2 follicles, theca cells from F3 follicles, granular cells from F3 follicles, theca cells from F4 follicles, granular cells from F4 follicles, and SFs). The result showed an extensive and negative relationship between the expression patterns of miRNA-458b-5p and *CTNNB1* (Figure 8).

## Discussion

Efficient ovarian development and ovulation is necessary for high-volume commercial egg production. Despite the identification of many genes and regulatory factors related to folliculogenesis and ovarian development, the molecular mechanisms and pathways underlying the transition from one developmental stage to the next remain unclear. Elucidating these will not only help us understand the program of ovarian follicular development, but will also provide better tools for chicken breeding.

The Jining Bairi chicken, an indigenous Chinese chicken breed, reaches sexual maturity more rapidly and lays earlier than commercial laying lines. Here, we present a systematic study of miRNA and mRNA profiles to analyze ovarian development in this breed. Four developmental stages are represented, using ovary tissue samples from sexually immature (60 days; stage A) (100 days; stage B), pre-laying (140 days; stage C), and laying/mature (140 days; stage D) animals.

**FIGURE 9:**
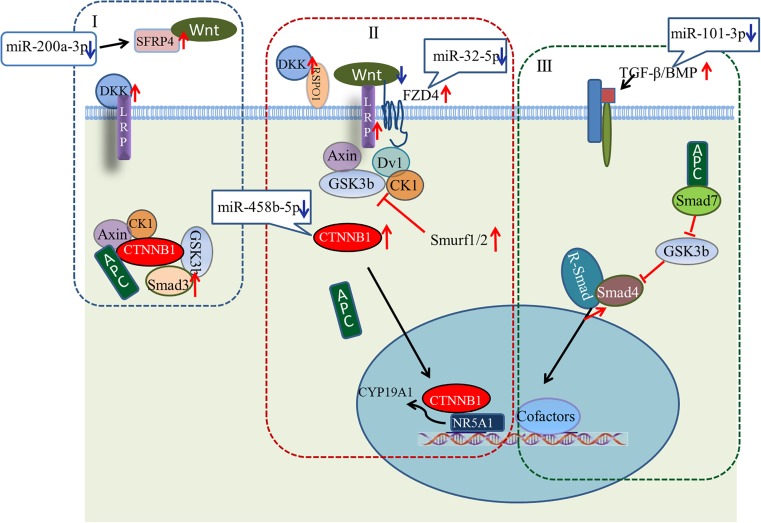
Model showing possible mechanism for regulation of ovarian development by four key miRNA/gene pairs. **(I)** Wnt/CTNNB1 signaling pathway in resting state; **(II)** Wnt/CTNNB1 signaling pathway is activated by the binding of a WNT to the FZD and LRP co-receptors; **(III)** TGF-beta pathway.

A large number of miRNA–mRNA interactions were predicted, including several experimentally identified pairs previously identified, such as miR-101-3p-*SLC38A2* and miR-200a-3p-*ERBB2IP* (Hafner et al., 2010). Notably, our analysis predicted follicular development-related pathway targets for many miRNAs. Hub nodes have been found to play important roles in many miRNA–gene networks (He and Zhang, 2006), and several miRNA hub nodes were identified. These include gga-miR-101-3p, gga-miR-200a-3p, gga-miR-32-5p, and gga-miR-458b-5p, which have high expression levels and are predicted to target several follicular development-related genes. Our study focused on these miRNAs and their targets, *BMP5*, *SFRP4*, *FZD4*, and *CTNNB1*. The functions of these genes within the context of the Wnt/TGF-beta pathway are summarized in Figure 9.

Wnt signaling first caught the attention of ovarian biologists after a landmark study by Vainio et al. (1999), which demonstrated that *WNT4* null mice lose most of their oocyte reserves in the days prior to birth. Subsequently, WNTs and Wnt signaling pathway components were found in the postnatal ovary, where many are differentially expressed throughout follicle development, suggesting potential roles in processes such as follicle formation, growth, and ovulation/luteinization (Hsieh et al., 2005; Parma et al., 2006; Chassot et al., 2008; Harwood et al., 2008; Hernandez et al., 2009). Many components of the Wnt/CTNNB1 pathway (one of three Wnt signaling pathways) are expressed during follicle development (Ricken et al., 2002; Hernandez-Gonzalez et al., 2006; Harwood et al., 2008; Wang et al., 2009). As shown in Figure 9, the linchpin of the canonical pathway is CTNNB1, a subunit within a large multiprotein complex that also includes the scaffold proteins APC and AXIN (Giles et al., 2003; Logan and Nusse, 2004). When bound to these proteins, CTNNB1 is phosphorylated by other components of the complex, resulting in subsequent ubiquitination and degradation (Figure 9I). The Wnt/CTNNB1 signaling pathway is activated by the binding of a WNT to the FZD and LRP co-receptors (Figure 9II), engendering the destruction of the complex and allowing CTNNB1 to escape and translocate to the nucleus. In granulosa cells, CTNNB1 enhances the expression of the *CYP19A1* gene, the key mediator of estrogen biosynthesis, through direct interaction with the transcription factor NR5A1 (Parakh et al., 2006).

Crosstalk between the Wnt signaling pathway and TGF-beta family occurs at multiple points. Wnt signaling inhibits phosphorylation of a multiprotein complex (Figure 9I), leading to Smad stabilization (Fuentealba et al., 2007). In response to stimulation by Wnt, the canonical Wnt and Smad pathways can synergize to activate transcription of target genes. Smad3 facilitates CTNNB1 nuclear translocation and coordinates with CTNNB1 at specific promoter sequences to regulate gene expression. Other components of the TGF-beta pathway, including Smurf1, Smurf2, and Smad7, modulate the Wnt signaling pathway (Luo, 2016). While the details of the canonical Wnt signaling and TGF-beta pathways are well understood in many cell types, whether the signal occurs *in vivo* as described, and whether these pathways are regulated by miRNAs during ovarian development, remain unresolved questions.

Our study predicted a negative relationship between gga-miR-200a-3p and *SFRP4*. *SFRP4* is a modulator of Wnt signaling that functions by binding WNTs directly and preventing their interaction with FZDs (Figure 9I). Based on our observations, we hypothesize that decreased levels of gga-miR-200a-3p in stage D ovaries enhance the expression of *SFRP4*. Although the role of *SFRP4* in the ovary is unclear, it may be involved in modulating follicle cell survival (Drake et al., 2003). Similarly, levels of gga-miR-32-5p and gga-miR-458b-5p decreased dramatically in stage D, consistent with the increased expression of *FZD4* and *CTNNB1*. *FZD4* has been detected throughout follicle development (Hsieh et al., 2002; Ricken et al., 2002). In addition, expression of the putative gga-miR-101-3p target *BMP5* increased in stage D ovaries. In this case, decreasing miRNA levels may help boost the Wnt signaling pathway. The increased expression of *LRP* and *DKK* favors this model, and further examination of these miRNA/mRNA pairs is warranted.

To conclude, deep sequencing of the transcriptome and microRNAome of the chicken ovary at four developmental stages reveals significant shifts in miRNA and mRNA expression. We identified 3166 putative miRNA-mRNA regulatory pairs containing 84 DEMs and 1047 DEGs. These are involved in various processes, including the Wnt signaling and the TGF-beta pathways. Understanding their relevance to ovarian development will be the focus of future research.

## Materials and Methods

### Animals

One hundred one-day-old Jining Bairi chickens were obtained from Jining Datang Chicken Breeding Co., Ltd. The chickens were raised under uniform environmental conditions at the conservation farm of the Shandong Agricultural University. Randomly selected individuals were sacrificed at 60 days (stage A), 100 days (stage B), 140 days but not yet laying eggs (stage C), and 140 days with egg laying having commenced (stage D). Eight chickens were sacrificed at each stage, of which four were used for miRNA and mRNA Illumina sequencing, and four for validation of the high-throughput sequencing data. Whole ovaries from stage A, B and C hens were collected. For stage D hens, follicles of different sizes, F1–F6 (10–34 mm in diameter), Y (yellow follicle, 6–8 mm in diameter), and W (white follicles, 2–4 mm in diameter), as well as the remaining ovarian tissues were collected. For large follicles, yolks were carefully removed. After being quick-frozen in liquid nitrogen, these tissues were ground into powder for isolation of total RNA.

### Library Construction and Deep Sequencing

Eight RNA libraries (four for sRNAs, and four for mRNAs) were constructed using pooled RNA from each stage (A, B, C, and D). For all libraries, total RNA (3.0 μg for each sample) was extracted using TRIzol Reagent (Invitrogen, Carlsbad, United States) according to the manufacturer’s protocol. The concentration and quality of RNA were determined using a NanoPhotometer^®^ spectrophotometer (IMPLEN, CA, United States) and an Agilent 2100 Bioanalyzer (Agilent Technologies, Palo Alto, CA, United States) (Supplementary Figure S6).

Libraries were prepared according to Illumina’s instructions using the NEBNext^®^ Multiplex sRNA Library Prep Set for Illumina^®^ (NEB, United States) or the NEBNext^®^ Ultra^TM^ RNA Library Prep Kit for Illumina^®^ (NEB, United States).

### Identification of miRNAs and mRNAs

After trimming adaptor sequences and removing contaminated reads, clean miRNA reads classified using computational methods. In brief, the clean reads were mapped to the NCBI chicken genome galGal4 with BowTie (Langmead et al., 2009). Reads corresponding to rRNA, tRNA, snRNA, scRNA, and snoRNA, identified using RepeatMasker and the Rfam database, were discarded. Novel miRNAs were predicted using miRDeep2 (Friedlander et al., 2012) and miREvo (Wen et al., 2012). Normalized miRNA reads were quantitated as TPM (Zhou et al., 2010). mRNA sequence reads were aligned to the chicken genome assembly version galGal4 using TopHat v2.0.9 with default parameters. Sequence segments were spliced and annotated using Cufflinks v2.1.1. Gene expression was quantitated as RPKM using HTSeq v0.5.4p3 and the Ensemble database as a reference (Mortazavi et al., 2008).

miRNA and mRNA raw counts were further normalized as TMM (trimmed Mean of M-values). DE analysis of miRNA and mRNA sequence data was performed with the Bioconductor package DEGseq R (Wang et al., 2010), using parameters *P* < 0.01 and |log_2_(fold change)| > 1.

### Quantitative Real-Time PCR

Total RNA was extracted using TRIzol Reagent and reverse transcribed using the One Step PrimeScript^®^ miRNA cDNA Synthesis Kit (Tiangen Biotech Co., China) according to the manufacturer’s instructions. The quality of RNA samples for qRT-PCR were detected by 1% agarose gel electrophoresis (Supplementary Figure S7). Real-time quantitative PCR was performed using an Mx3000p^TM^ SYBR^®^ Green Real-time quantitative PCR Analyzer (Stratagene, United States). The primer sequences for qRT-PCR are shown in Supplementary Table S12. The relative amounts of miRNA and mRNA were normalized against U6 snRNA and *GAPDH*, and the fold change for each miRNA and mRNA was calculated using the 2^-ΔΔCt^ method.

### Bioinformatic Analyses

GO term analyses and KEGG enrichment analyses for DEGs and target DEGs were performed using the R packages “clusterProfiler” (Yu et al., 2012) and “GOstats” (Falcon and Gentleman, 2007), with *p*-values calculated using right-sided hypergeometric tests. To prevent high false discovery rate (FDR) in multiple testing, *q*-values were also estimated for FDR control (Storey, 2003). Figures were prepared using the R packages “ggplot2” (Wilkinson, 2011) and ‘UpSetR’ (Gehlenborg, 2016).

Identification of miRNA–mRNA regulatory relationships was based on computational target predictions and negative regulation relationships. TargetScan and miRanda were used for computational target prediction. An R script (miRCausality.R) was used to identify miRNA-mRNA regulatory relationships (Le et al., 2013). The method learns a causal structure from expression data, and applies *do-calculus* to infer regulatory effects (reported over the range -1 to 1). We calculated pairwise effects between each DE miRNA and mRNA based on their expression across all samples. A regulatory effect less than -0.9 was considered to represent a negative regulatory relationship. Cytoscape was used to visualize and analyze the miRNA–mRNA networks, and network topology was analyzed using the NetworkAnalyzer Cytoscape plugin (Assenov et al., 2008).

## Ethics Statement

This study was carried out in accordance with the recommendations of Guidelines for Experimental Animals established by the Ministry of Science and Technology (Beijing, China). The protocol was approved by the Institutional Animal Care and Use Ethics Committee of Shandong Agricultural University.

## Availability of Data

The datasets for this study can be found in the NCBI SRA database under accession number SRP130184 (https://www.ncbi.nlm.nih.gov/sra/SRP130184).

## Author Contributions

HT, QZ, WW, and KW conceived this study and carried out the data analyses and wrote the manuscript. LK and SS performed the library construction and Illumina sequencing. MJ conducted the experimental validation. All authors reviewed and approved the final manuscript.

## Conflict of Interest Statement

The authors declare that the research was conducted in the absence of any commercial or financial relationships that could be construed as a potential conflict of interest.

## Abbreviations

BMP15: bone morphogenetic protein 15
CCND2: cyclin D2
CYP11A1: cytochrome P450, family 11, subfamily a, polypeptide 1
DEGs: differentially expressed genes
DEMs: differentially expressed miRNAs
FSH: follicle-stimulating hormone
FSHR: FSH receptor
GDF9: growth differentiation factor-9
GO: gene ontology
KEGG: Kyoto Encyclopedia of genes and genomes
LW: large white follicle
miRNAs: microRNAs
RPKM: reads per kilo bases per million
SAV1: salvador homolog
SF: small yellow follicle
sRNA: small RNA
STAR: steroidogenic acute regulatory protein
TPM: transcripts per million
