# Supplementary material for: Dynamic Changes in the Global MicroRNAome and Transcriptome Identify Key Nodes Associated With Ovarian Development in Chickens
Source: Front Genet. 2018 Oct 23;9:491. doi: 10.3389/fgene.2018.00491 (PMC6206165; doi:10.3389/fgene.2018.00491)

Figure S1 The length distribution of the small RNA in four miRNA libraries.

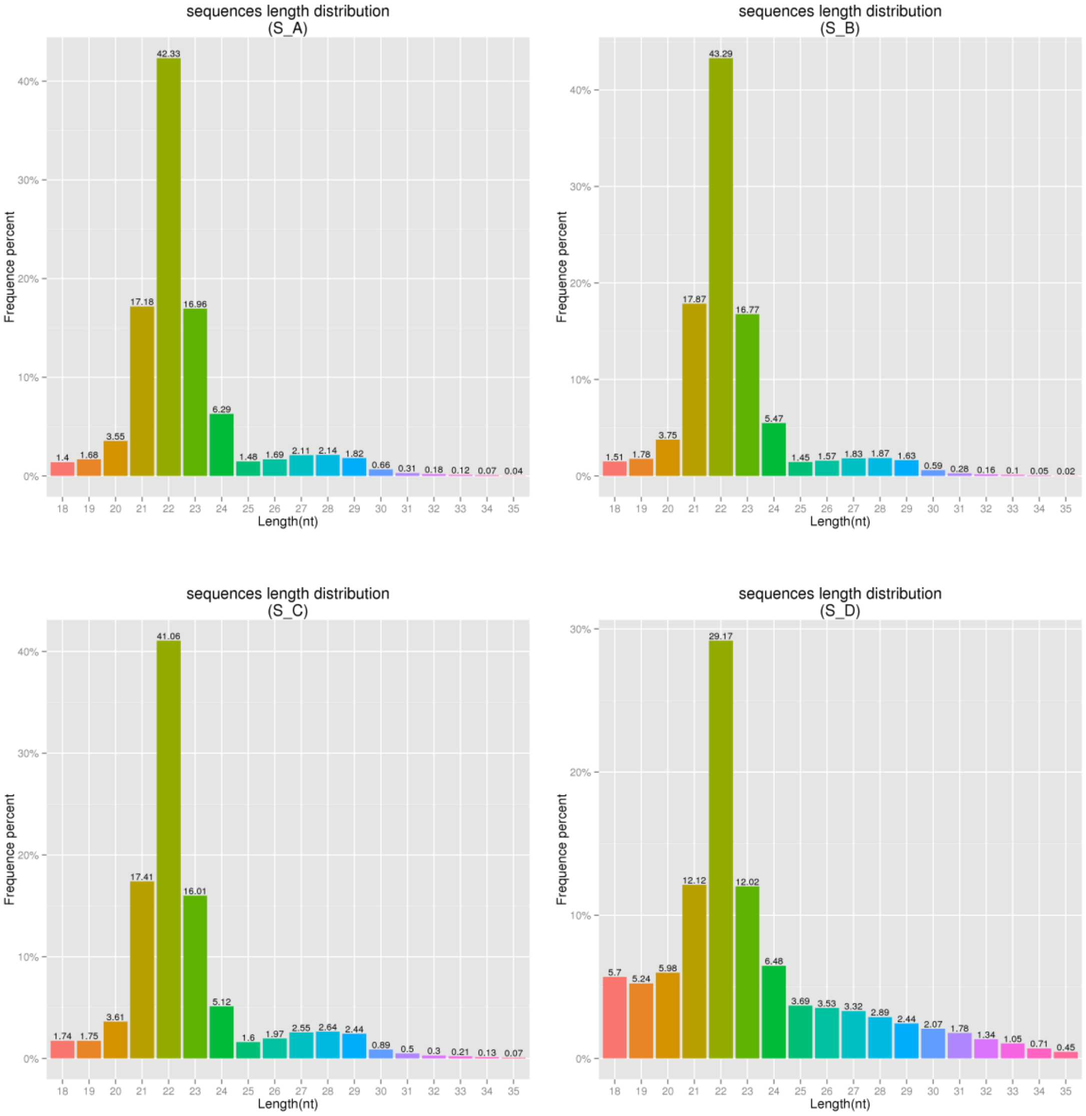

Figure S2 The distribution of reads on chromosomes.

S_A

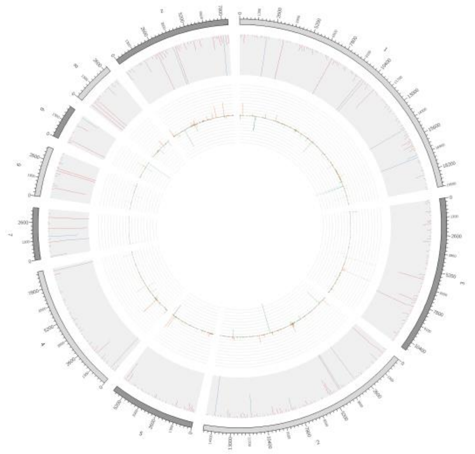

S_B

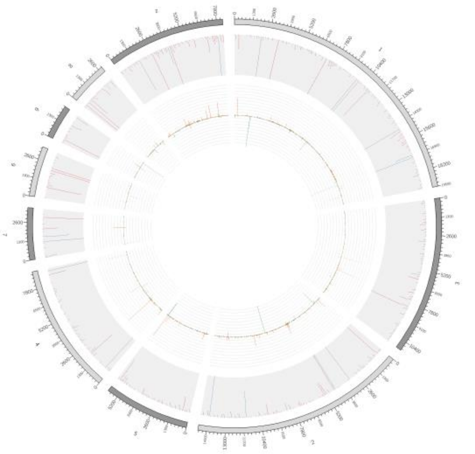

S_C

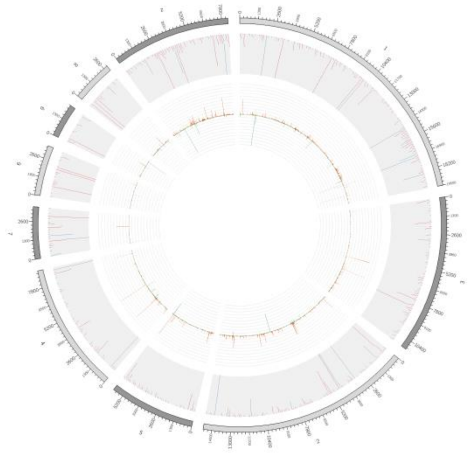

S_D

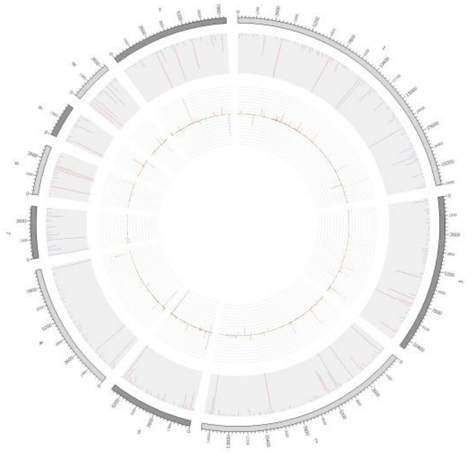

Figure S3 The volcano maps of differently expressed miRNAs.

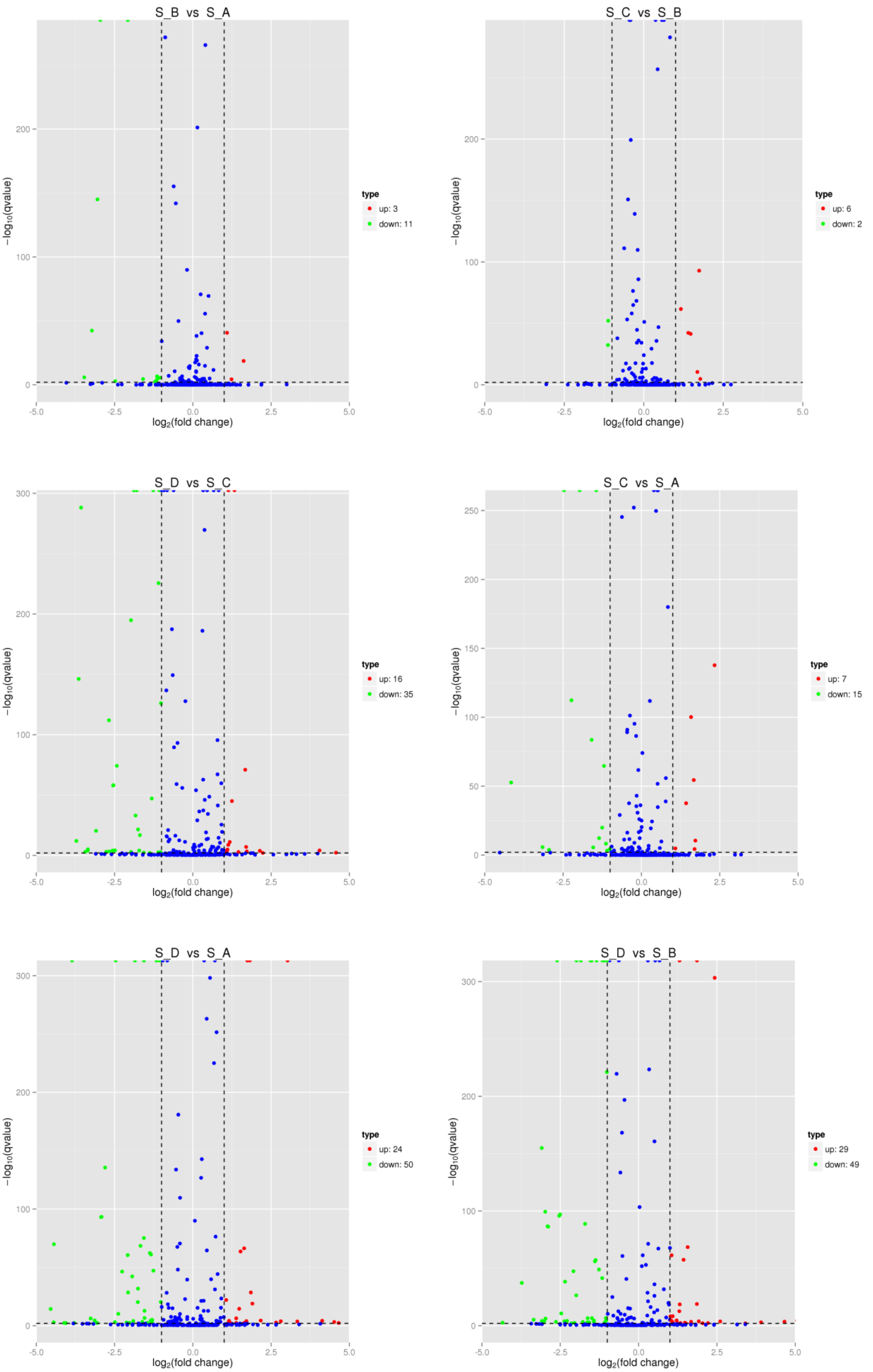

Figure S4 The reads distribution of four cDNA libraries.

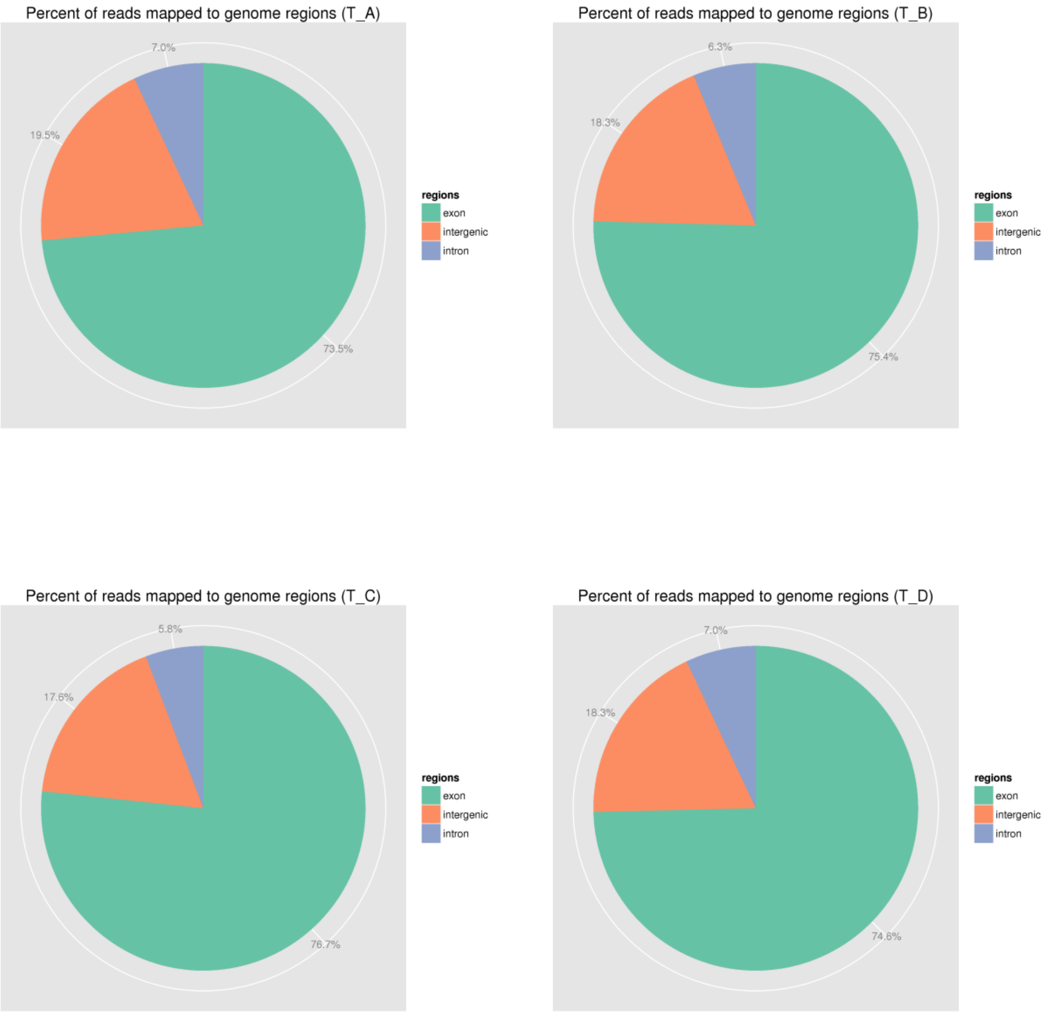

Figure S5 The volcano maps of differently expressed mRNAs.

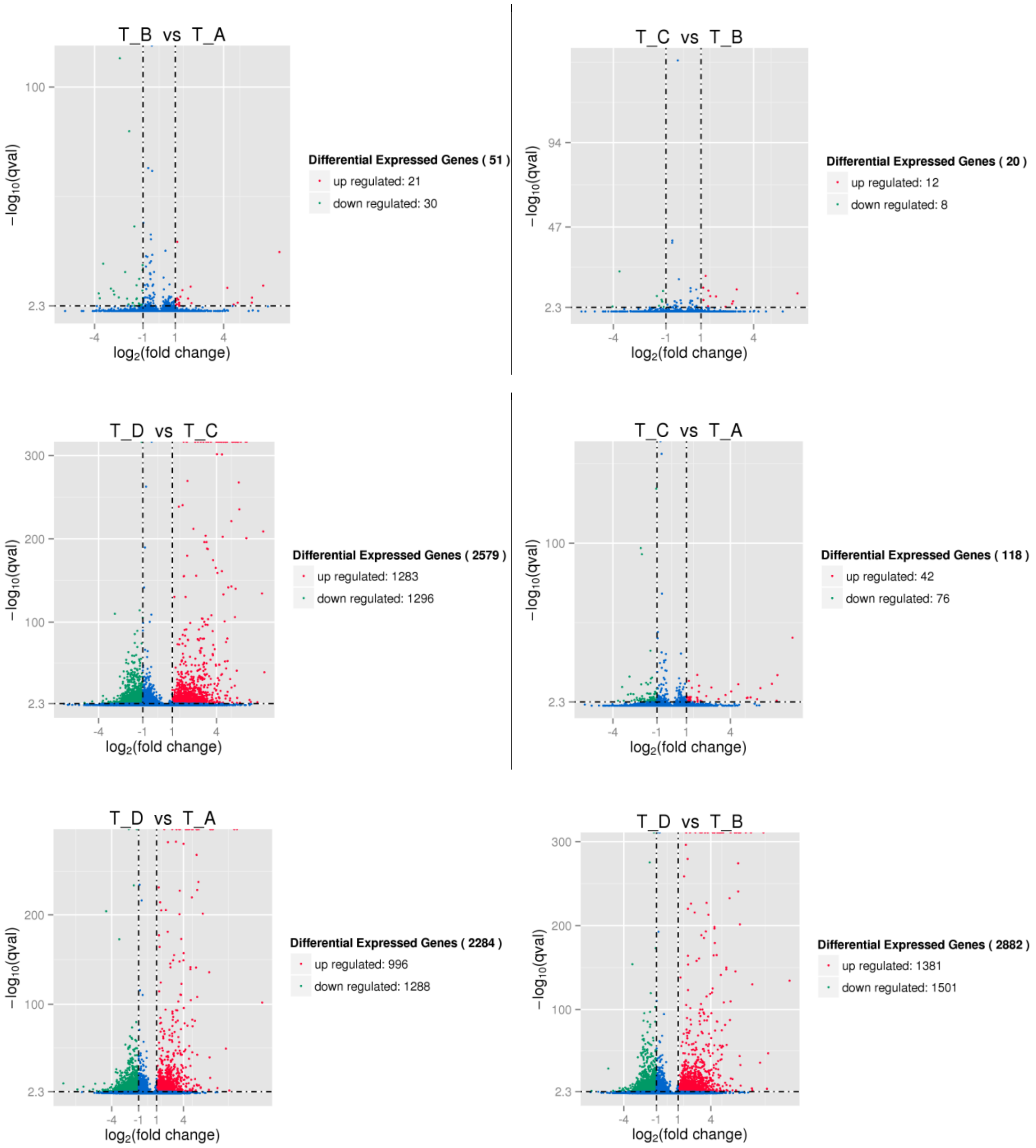

Figure S6 The details of RNA quality determined using an Agilent 2100 Bioanalyzer.

S_A

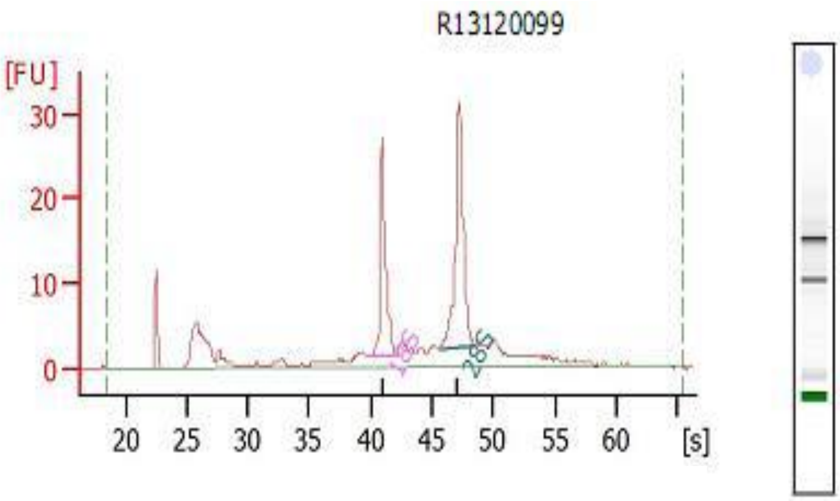

RNA Area:	206.2
RNA Concentration:	101 ng/µl
rRNA Ratio [28s / 18s]:	1.4
RNA Integrity Number (RIN):	8.9 (B.02.08)
Result Flagging Color:	
Result Flagging Label:	RIN: 8.90

S_B

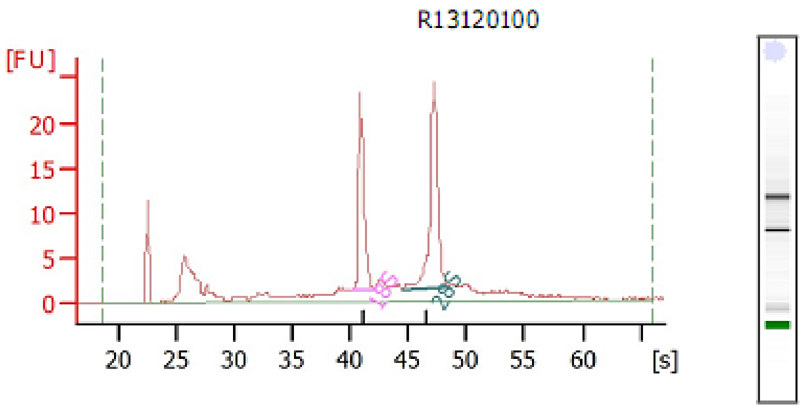

RNA Area:	159.6
RNA Concentration:	78 ng/µl
rRNA Ratio [28s / 18s]:	1.5
RNA Integrity Number (RIN):	8.9 (B.02.08)
Result Flagging Color:	
Result Flagging Label:	RIN: 8.90

S_C

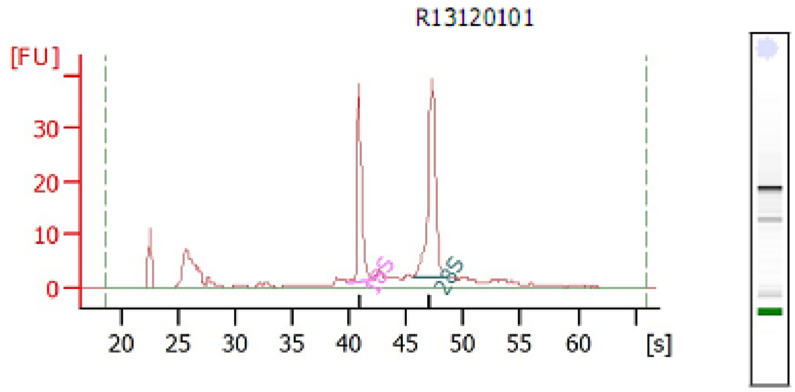

RNA Area:	209.0
RNA Concentration:	102 ng/µl
rRNA Ratio [28s / 18s]:	1.7
RNA Integrity Number (RIN):	9.4 (B.02.08)
Result Flagging Color:	
Result Flagging Label:	RIN: 9.40

S_D

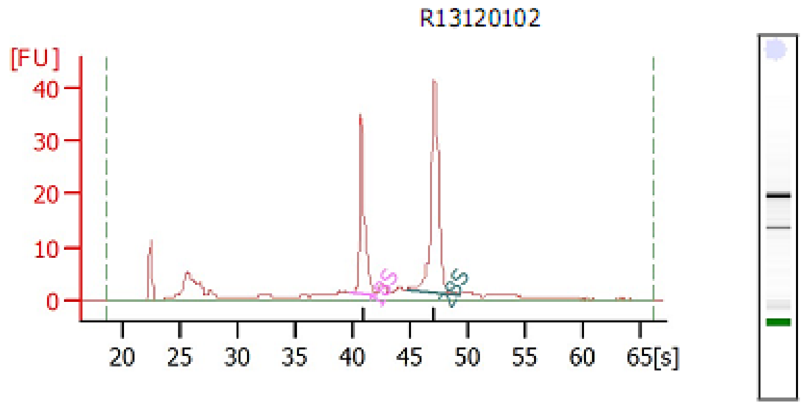

RNA Area:	206.0
RNA Concentration:	101 ng/µl
rRNA Ratio [28s / 18s]:	1.8
RNA Integrity Number (RIN):	9.4 (B.02.08)
Result Flagging Color:	
Result Flagging Label:	RIN: 9.40

Figure S7 The quality of RNA samples detected by 1% agarose gel electrophoresis.

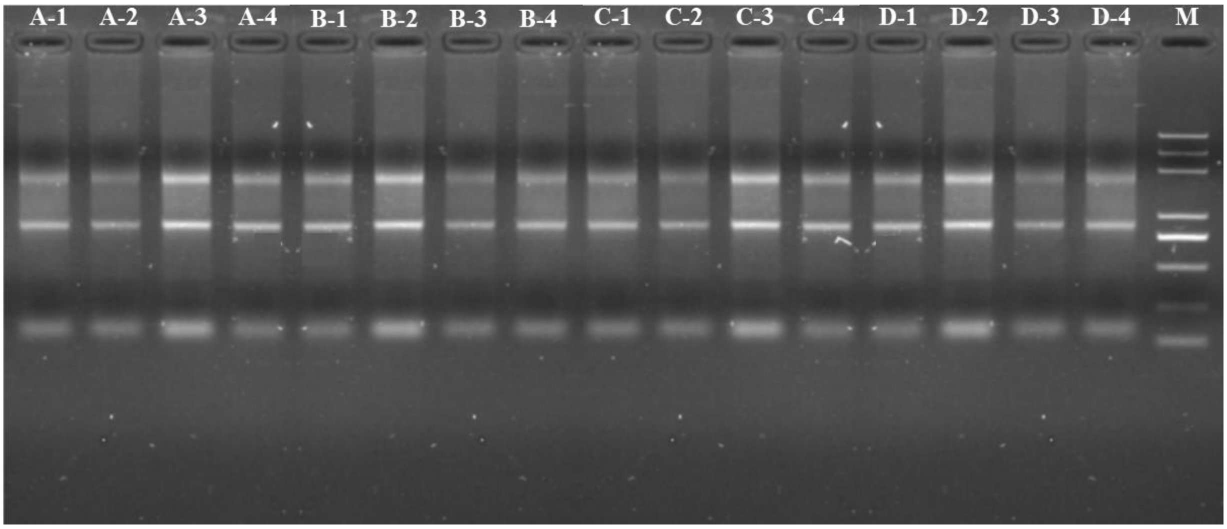

Supplement: Supplementary file 1 [file Image_1.PDF]
